# The potential impact of HPV-16 reactivation on prevalence in older Australians

**DOI:** 10.1186/1471-2334-14-312

**Published:** 2014-06-06

**Authors:** Igor A Korostil, David G Regan

**Affiliations:** 1 The Kirby Institute, University of New South Wales, 2052 Sydney, Australia

**Keywords:** HPV latency, Vaccination, Reactivation

## Abstract

**Background:**

Some regional cross-sectional human papillomavirus (HPV) DNA prevalence data show an increase in prevalence in older women, the reasons for which are as yet unknown. A recently published study suggests that the increase may be at least partly due to reactivation of latent HPV in menopausal women.

**Methods:**

We developed a dynamic mathematical model of HPV-16 transmission to estimate the key consequences of hypothetical HPV-16 reactivation in the Australian heterosexual population. We only consider a worst case scenario with regard to reactivation in the Australian setting when all women who are latently infected reactivate and, wherever feasible, we choose model parameter values which may lead to a more pronounced reactivation. The ongoing National HPV vaccination program covering both women and men is incorporated in the model.

**Results:**

We estimate that about 1 in 10 women and men who appear to have cleared HPV-16 infection may be latently infected. The prevalence of HPV-16 in older Australian women will increase by a factor of up to 3.1 between now and 2025 which will be accompanied by an increase by a factor of around 1.9 in older men. However, the long-term impact of the HPV vaccination is not significantly altered by reactivation.

**Conclusions:**

If the reactivation hypothesis we consider is substantiated, the public health response should be focused on further improvement of cervical screening coverage for older women. Our study also highlights the urgent need for surveillance of HPV prevalence in older Australians.

## Background

In general, HPV DNA prevalence peaks in women aged 20–24 and then steadily declines with age. However, in some regions the HPV age-specific prevalence curve has been observed to be U-shaped, i.e., exhibiting an increase in prevalence in older women (see [1-3] for detailed reviews). We do not know whether this is the case in Australia, as the only local prevalence data were obtained by the Womens HPV Indigenous Non-Indigenous Urban Rural Study (WHINURS) [4] which only enrolled women aged 15 to 39.

It has been hypothesised that reactivation of HPV may be at least partly responsible for the high HPV prevalence observed in older women (see, for example, [5,6]), while another possibility worth consideration is changes in sexual behaviour of men and/or women in middle-age [1]. In the case of Australia, the latter appears unlikely to be responsible for a U-shaped prevalence curve, should it be observed, as the most recently conducted comprehensive sexual behaviour survey (the Australian Study of Health and Relationships (ASHR)), did not demonstrate any notable increase in sexual activity in older Australians [7].

Results from a recently published study by Gravitt et al. lend plausibility to the hypothesis of HPV reactivation in menopausal women [8], which may be happening due to the hormonal changes altering immune function (related findings in [9] are of interest in this context). These findings may be interpreted as indicating that any menopausal woman who has ever been infected with HPV-16 is at risk of reactivation. In addition, the study by Gravitt et al. speculates that a cohort effect associated with the sexual revolution may have masked the second peak in the age-specific HPV prevalence curves of some Western countries. Briefly, the idea is that due to the sexual revolution and the subsequent increase in sexual activity and HPV-16 prevalence (as compared with pre-sexual revolution times), a cross-sectional study collecting HPV-16 prevalence data in the early 2000s would cover younger women with post-sexual revolution sexual experiences and older women whose sexual behaviour had been defined by the pre-sexual revolution norms. While there would be HPV-16 reactivation in these older women, it would result in HPV-16 prevalence lower than that in the younger women from the post-sexual revolution cohort. Therefore, age-specific HPV-16 prevalence curves would show no signs of HPV reactivation. However, reactivation may be detected by prospective studies conducted when the women who became sexually active after the sexual revolution reach menopause.

Assuming that the above is indeed valid, we are interested in clarifying if the issue of HPV-16 reactivation should be seen as important for the Australian public health. To address this question, we model HPV-16 transmission in the Australian heterosexual population implementing reactivation of the virus in a manner we consider to be plausible based on our current knowledge. We only consider the worst case scenario, whereby all women at risk of reactivation do reactivate and vaccine efficacy is set at the lower plausible bound. We estimate the proportion of women at risk of reactivation, the impact on HPV-16 prevalence in older women and older men as a result of reinfection of susceptible men within existing partnerships. We also compare the long-term reductions in HPV-16 prevalence due to the ongoing Australian HPV vaccination program produced by the versions of our model with and without reactivation.

## Methods

Our model is briefly described below with a complete description, including justification of assumptions, provided in Additional file 1: Technical Appendix.

### Model structure and assumptions regarding HPV-16 reactivation

**Figure 1:**
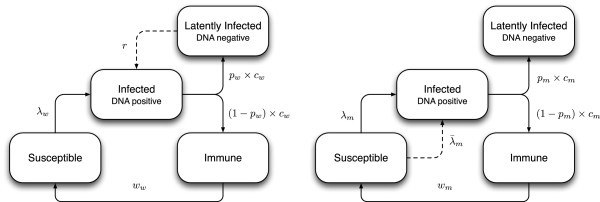
**Schematic diagram of the HPV-16 transmission model for females (left) and males (right).** Here *λ*_*w*_ and *λ*_*m*_ are the forces of infection (i.e. the rates of becoming infected) for women and men, ${\bar{\lambda }}_{m}$ is an additional force of infection acting on older men whose partners become infected via reactivation, and other parameters are as in Table 1.

We developed a dynamic compartmental mathematical model of HPV-16 transmission in the Australian heterosexual population. The model is illustrated schematically in Figure 1. This model is essentially of SIRS (Susceptible-Infected-Removed (Immune)-Susceptible) structure with waning natural immunity [10], extended to incorporate the possibility that HPV-16 may enter a latent undetectable state and subsequently reactivate (in women). The SIRS structure, while not the only plausible representation of HPV natural history [11], was chosen because it has been the most widely used (see, for example, [12] for a review). This structure has the advantage that when natural immunity wanes very slowly, the model approximates the SIR structure (life-long immunity) and if waning occurs very quickly, it approaches the SIS structure (no immunity). Our model incorporates both reactivation and reinfection, and we do not impose *a priori* the extent to which either should prevail. Susceptible women and men in the model may become infected and then some of them clear the infection completely and become immune, while others enter a latent infected state in which it is assumed that they would not test DNA positive due to viral load falling below the detectable threshold.

**Table 1:** Model parameters, their prior distributions (all uniform), posterior means and standard deviations (SD)

**Model**		**Prior**		**Posterior**	
**Parameter**	**Symbol**	**Specification**	**Refs.**	**Mean**	**SD**
			Natural history parameters		
Probability of HPV-16 transmission					
From female to male	*β*_*w* *m*_	*U*(0.10,1.00)	[25]	0.92	0.03
From male to female	*β*_*m* *w*_	*U*(0.10,1.00)	[25]	0.93	0.03
Rate of clearance of HPV-16					
For women under 30	*c*_*w*1_	*U*(0.50,1.17)	[26-29]	0.59	0.05
For women over 30	*c*_*w*2_	*U*(0.50,1.17)	[26-29]	0.60	0.05
For men under 30	*c*_*m*1_	*U*(0.55,1.66)	[30,31]	0.62	0.03
For men over 30	*c*_*m*2_	*U*(0.55,1.66)	[30,31]	0.63	0.03
Probability of becoming latently infected					
For women	*p*_*w*_	*U*(0.01,1.00)	n/a	0.11	0.04
For men	*p*_*m*_	*U*(0.01,1.00)	n/a	0.09	0.05
Rate of loss of immunity					
For women under 30	*w*_*w*1_	*U*(0.01,2.00)	n/a	1.65	0.16
For women over 30	*w*_*w*2_	*U*(0.01,2.00)	n/a	1.67	0.15
For men under 30	*w*_*m*1_	*U*(0.01,2.00)	n/a	1.78	0.14
For men over 30	*w*_*m*2_	*U*(0.01,2.00)	n/a	1.82	0.10
			Sexual behaviour parameters		
Degree of assortativity					
By age group	*ε*_*a*_	*U*(0.05,0.95)	n/a	0.27	0.22
By sexual activity group	*ε*_*s*_	*U*(0.05,0.95)	n/a	0.64	0.21

Note that all parameters denote quantities averaged over the modelled population; the degrees of assortativity *ε*_*a*_ and *ε*_*s*_ are implemented as described in [32]; n/a indicates that there is no published literature to inform the choice of prior distribution and the values given are based on assumption. The choice of prior distributions is discussed in detail in Additional file 1: Technical Appendix.

The following key assumptions have been made: 

• Any woman infected with HPV-16 at least once can potentially become latently infected [8];

• All women with latent HPV-16 infection reactivate at menopause at constant age-specific rates equal to the rates of onset of menopause;

• Latently infected men and women are not infectious;

• Men can be latently infected in which case they are not infectious, but they can not reactivate and cannot be reinfected;

• The onset of menopause occurs in women aged 45–54 [13,14]

• 70% of all Australian women aged 45 to 54 are assumed to be in the long-term partnerships [15]; this is important because the susceptible partners of these women can become infected without entering any new sexual partnerships.

### Assumptions regarding sexual behaviour

We assume that sexual activity in the Australian heterosexual population increased substantially during the sexual revolution of 1961–1975 and then stabilised at a level close to that described by the ASHR study in 2001–2002. This is a necessary simplification primarily based on public perception and backed by some sparse evidence (see [16] for a detailed discussion). We also assume that sexual behaviour as captured by ASHR will not change substantially over the next 50 years or so. This may soon prove to be an unrealistic assumption in view of the recent data suggesting that sexually active young people have an increasing number of sexual partners [17].

### Assumptions regarding vaccination

The vaccine is assumed to be prophylactic (i.e., not therapeutic and only protecting susceptible individuals from infection), and the duration of vaccine induced protection, implemented in the model as reduced susceptibility to reinfection, is assumed to be life-long. Vaccine coverage was fixed at 72% for 12 year old girls (based on 3-dose coverage achieved thus far under the Australian vaccination program [18,19]), and at 72% for boys (since they are vaccinated at schools along with girls, there is currently no reason to believe their coverage will be substantially less than in females). For the 2007–2009 catch-up vaccination the achieved 3-dose coverage over two years was 72%/ 72%/ 62%/32% for women aged 13–15/16–17/18–19/20–26. For the catch-up campaign for 14–15 year old boys the 3-dose coverage was assumed to be 72% equally split between 2013 and 2014. While the actual efficacy of the vaccine at preventing transmission is still unknown, we assumed the lower bounds of 80% for women [20,21] and 50% for men based on data from the clinical trials on efficacy against persistent infection [22]. The same trials showed that the efficacy could also be as high as 90% in women and 80% in men, but we consider the worst case scenario in this paper. For simplicity, and because it seems plausible, we assume that vaccinated women are not at risk of reactivation (see [23] for discussion).

### Model calibration

The model was calibrated to the HPV-16 age-specific prevalence in non-Indigenous Australian women aged 15–39 as reported in the WHINURS study [4]. These data were collected in 2005–2007, hence they describe the prevalence immediately before the commencement of the National HPV vaccination program in 2007. HPV-16 prevalence data for men are not available for Australia. We applied a Bayesian methodology detailed in [24] which entails the specification of prior probability distributions for model parameters and these are listed in Table 1.

## Results

**Figure 2:**
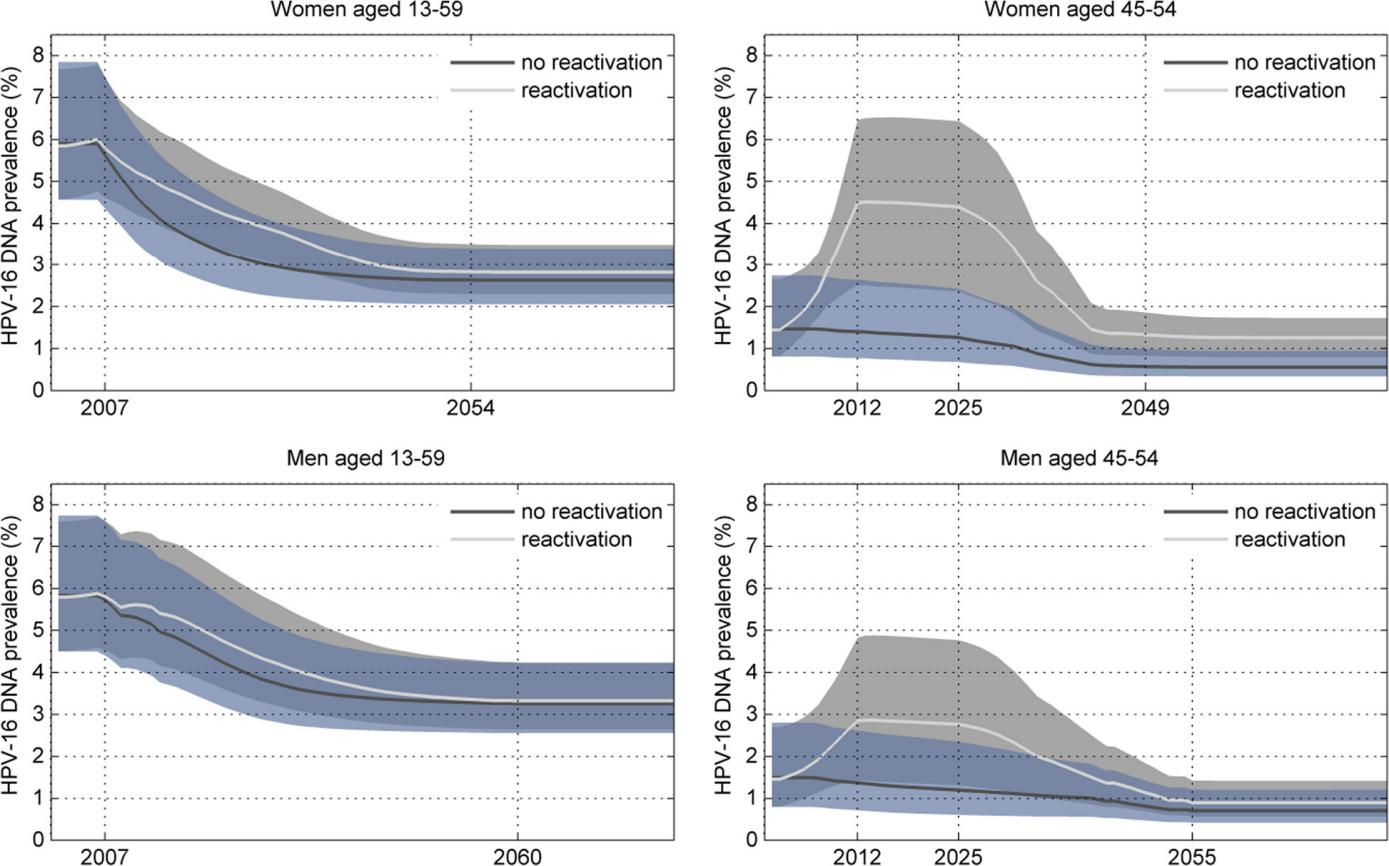
**Temporal changes in HPV-16 DNA prevalence in men and women predicted by the model with and without reactivation in menopausal women.** Solid lines are means and 95% confidence intervals are shown as shaded areas between the 2.5-th and 97.5-th percentiles. The panels on the left-hand side show the overall prevalence in the sexually active male and female populations. The panels on the right-hand side show prevalence in the older male and female populations where the effect of reactivation is much more pronounced.

The means and standard deviations of the inferred parameter distributions (i.e., posterior distributions) obtained through the calibration process are shown in Table 1 and a brief description of the key observations follows. The mean per-partnership transmission probabilities for male-to-female transmission, *β*_*m* *w*_, and for female-to-male transmission, *β*_*w* *m*_, inferred by the model are nearly equal (0.93 and 0.92, respectively). It should be noted, however, that these are usually overestimated by compartmental models [33]. The inferred rates of clearance are marginally higher for older men and women (as compared with the younger men and women). The differences in these rates are insufficient to contradict empirical estimations where the rates are not dependent on age [27,29], though they conflict with the higher rates for younger women reported in [28]. On the other hand, our estimations are consistent with findings from a large study in men which observed that older men tended to clear HPV notably quicker than younger men, which might be attributable to a higher prevalence of HPV antibodies in older men [22]. The probability (per infection) of becoming latently infected is estimated to be at around 0.1 for both men and women.The effect of HPV-16 reactivation in terms of temporal changes in HPV-16 DNA prevalence in the Australian setting is illustrated in Figure 2. The model predicts that the total sexually active male population experiences a small overall increase in HPV-16 prevalence, but in older men prevalence increases more substantially by a factor of around 1.9 by the end of 2012 (since when the time of sexual debut for all 45–54 year old women is after the sexual revolution). By the same year prevalence in older women is predicted to increase by a factor of around 3.1. A steady decrease in prevalence begins after 2025 as women aged 26 who were covered by the catch-up vaccination in 2007 turn 45. By 2050, 72% of women aged 45–54 will have been vaccinated, so afterwards prevalence in these women is largely stabilised. After 2055, 72% of men and women aged 45–54 are vaccinated, and 72% of the entire modelled population is vaccinated by the end of 2060. From this point onwards, HPV-16 prevalence, reflecting the long-term impact of vaccination, is steady for all subpopulations. Our results suggest that the prevalence achieved by vaccination under the assumption of reactivation as compared to no reactivation is visibly higher only in older women, where it is sustained by unvaccinated women reactivating at menopause (these will comprise 28% of all women aged 45–54). In older men, reactivation will directly affect only those who remain unvaccinated (28% of all older men) and susceptible while being in long-term partnerships with unvaccinated older menopausal women. The proportion of such men will be insignificant, hence the barely distinguishable difference between prevalence predicted with and without reactivation shown in Figure 2.

## Discussion

In this paper, we present a simple model-based evaluation of the potential impact of reactivation of HPV-16 in Australian menopausal women in terms of changes in HPV-16 prevalence. This is necessary for any further analysis regarding progression to cervical and other cancers in both women and men. We focus on the strongest impact plausible given our current limited understanding of how reactivation may work and because from the public health perspective evaluation of the worst case scenario is essential to assess the scale of hypothetical reactivation issue in principle. The model we set up is adequate for our purposes at this stage, when we effectively aim to outline the magnitude of the effect reactivation brings into a model not accounting for its possibility. While intuition suggests what should generally happen to HPV-16 prevalence in Australia if reactivation occurs (increase in older women, less substantial increase in older men), mathematical modelling helps to quantify this. In particular, there is no means to estimate the proportion of latently infected individuals except modelling.

Our results suggest that the long-term effect of reactivation will be insignificant as at the post-vaccination equilibrium only 28% of all women will be unvaccinated and so, theoretically, at a risk of reactivation, but according to our estimations only about 1/10 of these may become latently infected. However, we are more interested in the period when older women in the modelled population are largely unvaccinated, which is roughly between now and early the 2030s. We conclude that reactivation has important implications for predicting the impact of vaccination in the short to medium term and for surveillance and screening activities. The fact that from 2013 HPV-16 prevalence in older women is predicted to nearly triple under the reactivation assumption as compared to its pre-reactivation level is a cause for concern. Similarly, the almost 60% increase predicted in older men is of concern, since unlike women who are covered by the National Cervical Screening Program (NCSP) [34], men are not screened. Should sufficient clinical evidence for HPV reactivation become available in the near future, the NCSP may have to consider means to improve screening coverage among older women. It is not clear what measures may be taken in response to the increased prevalence in older men who would be at an increased risk of developing HPV-16 associated cancers (anal, penile, throat cancers) later in their lives. Residual life expectancy for men in Australia has been constantly growing: it reached 17.6 years for those aged 65 in 2003 [35]. Hence, the issue of cancers in older men can not be dismissed due to a high likelihood of them dying in their mid-60s.

It is important to mention some limitations of our study. Among the numerous assumptions we have made (see Additional file 1: Technical Appendix for discussion), a special role is played by those regarding sexual behaviour. Our statement regarding a stable sexual behaviour after sexual revolution should be understood as a necessary simplification due to the absence of historical sexual behaviour data. It is, however, acceptable. If, for example, sexual behaviour in the Australian population in response to AIDS declined in the 80s (similarly to what happened in the US [36]), there would be a cohort of menopausal women less exposed to HPV than that in our model. As our study aims to investigate the worst case scenario, simulating such a decline is not necessary. In addition, we implicitly assume that current sexual behaviour will remain roughly unchanged, but this is done in all modelling studies we are aware of as it is not possible to make robust predictions into the future. Considering only the heterosexual population may be an omission, as well as not considering the impact of temporary visitors, Australian travellers overseas and immigrants. The latter is unquestionably a subject of future research since Australia accepts high numbers of immigrants each year [37]. We did not implement the possibility of existence of immune memory [38] or clearly separate persistent and incident infections (as discussed in [28,39]). Finally, we should stress that our study should be seen as a preliminary evaluation of the potential impact of reactivation in Australia and is intended to estimate the worst increases in HPV-16 prevalence due to inclusion of reactivation in a transmission model simulating the National HPV vaccination program. However, as HPV reactivation remains a hypothesis, more comprehensive reactivation modelling studies do not yet appear timely.

## Conclusions

We do not expect our findings to serve as a basis for any immediate public health decision making. However, we hope that our results will draw the attention of the public health community to the subject of HPV reactivation and serve as motivation to conduct a study focusing on the collection of HPV-16 DNA prevalence data in older Australian women and, if feasible, men. This study would have to be conducted as soon as possible, as after 2025 its relevance in the Australian setting will diminish. Any clinical evidence for or against reactivation would contribute to more efficient detection and prevention of HPV, especially in countries where HPV vaccination programs have not yet reached their full potential.

## Competing interests

The authors declare that they have no competing interests.

## Authors’ contributions

DGR coordinated the study and participated in study design, model parameterisation and manuscript preparation. IAK conceived of the study, developed and implemented the models, analysed results and drafted the manuscript. All authors read and approved the final manuscript.

## Pre-publication history

The pre-publication history for this paper can be accessed here:

http://www.biomedcentral.com/1471-2334/14/312/prepub

## Supplementary Material

Additional file 1 Technical Appendix. Click here for file

## References

[B1] de Sanjosé S Diaz M Castellsagué X Clifford G Bruni L Muñoz N Bosch FX **Worldwide prevalence and genotype distribution of cervical human papillomavirus DNA in women with normal cytology: a meta-analysis** Lancet Infect Dis 2007 14 7 453 459 10.1016/S1473-3099(07)70158-5 17597569

[B2] Smith JS Melendy A Rana RK Pimenta JM **Age-specific prevalence of infection with human papillomavirus in females: a global review** J Adolesc Health 2008 14 4 Suppl S5 25– S25.e1–41 18809145 10.1016/j.jadohealth.2008.07.009

[B3] Bruni L Diaz M Castellsagué X Ferrer E Bosch FX de Sanjosé S **Cervical human Papillomavirus prevalence in 5 continents meta-analysis of 1 million women with normal cytological findings** J Infect Dis 2010 14 12 1789 1799 10.1086/657321 21067372

[B4] Garland SM Brotherton JM Condon JR McIntyre PB Stevens MP Smith DW Tabrizi SN group tWs **Human papillomavirus prevalence among indigenous and non-indigenous Australian women prior to a national HPV vaccination program** BMC Medicine 2011 14 104 10.1186/1741-7015-9-104 21910918 PMC3182900

[B5] Herrero R Castle PE Schiffman M Bratti MC Hildesheim A Morales J Alfaro M Sherman ME Wacholder S Chen S Rodriguez AC Burk RD **Epidemiologic profile of type-specific human papillomavirus infection and cervical neoplasia in Guanacaste, Costa Rica** J Infect Dis 2005 14 11 1796 1807 10.1086/428850 15871111

[B6] Strickler HD Burk RD Fazzari M Anastos K Minkoff H Massad LS Hall C Bacon M Levine AM Watts DH Silverberg MJ Xue X Schlecht NF Melnick S Palefsky JM **Natural history and possible reactivation of human papillomavirus in human immunodeficiency virus-positive women** JNCI J Nat Cancer Inst 2005 14 8 577 586 10.1093/jnci/dji073 15840880

[B7] **The Australian study of health and relationships web site** [http://www.ashr.edu.au] last accessed: 25 July, 2013

[B8] Gravitt PE Rositch AF Silver MI Marks MA Chang K Burke AE Viscidi RP **A cohort effect of the sexual revolution may be masking an increase in human papillomavirus detection at menopause in the United States** J Infect Dis 2012 14 2 272 280 23242540 10.1093/infdis/jis660 PMC3532829

[B9] Althoff KN Paul P Burke AE Viscidi R Sangaramoorthy M Gravitt PE **Correlates of cervicovaginal human papillomavirus detection in perimenopausal women** J Women’s Health 2009 14 9 1341 1346 10.1089/jwh.2008.1223 PMC2825723 19702476

[B10] Vynnycky E White RG An Introduction to Infectious Disease Modelling 2010 New York: Oxford University Press

[B11] Korostil IA Peters GW Law MG Regan DG **Herd immunity effect of the HPV vaccination program in Australia under different assumptions regarding natural immunity against re-infection** Vaccine 2013 14 15 1931 1936 10.1016/j.vaccine.2013.02.018 23434388

[B12] Garnett GP Kim JJ French K Goldie SJ **Chapter 21: Modelling the impact of HPV vaccines on cervical cancer and screening programmes** Vaccine 2006 14 S3/178 S3/186 10.1016/j.vaccine.2006.05.116 16950005

[B13] Do KA Treloar SA Pandeya N Purdie D Green AC Heath AC Martin NG **Predictive factors of age at menopause in a large Australian twin study** Hum Biol 1998 14 6 1073 1091 9825597

[B14] Guthrie J Dennerstein L Taffe J Lehert P Burger H **Hot flushes during the menopause transition: a longitudinal study in Australian-born women** Menopause 2005 14 4 460 467 10.1097/01.GME.0000155200.80687.BE 16037762

[B15] de Vaus D Diversity and Change in Australian Families. Statistical Profiles 2004 Melbourne: Australian Institute of Family Studies

[B16] Lewis MJ Thorns on the Rose: the History of STDs in Australia in International Perspective 1998 Canberra: Australian Govt. Pub. Service

[B17] Agius PA Pitts MK Smith AMA Mitchell A **Sexual behaviour and related knowledge among a representative sample of secondary school students between 1997 and 2008** Aust N Z J Public Health 2010 14 5 476 481 10.1111/j.1753-6405.2010.00593.x 21040175

[B18] Gertig DM Brotherton JML Saville M **Measuring human papillomavirus (HPV) vaccination coverage and the role of the National HPV Vaccination Program Register, Australia** Sex Health 2011 14 2 171 178 10.1071/SH10001 21592430

[B19] **National HPV vaccination program register web site** [http://www.hpvregister.org.au] last accessed: 13 May, 2014

[B20] Lazcano-Ponce E Lörincz AT Salmeron J Fernández I Cruz A Hernández P Mejia I Hernandez-Avila M **A pilot study of HPVDNA and cytology testing in 50,159 women in the routine Mexican Social Security Program** Cancer Causes Control 2010 14 10 1693 1700 10.1007/s10552-010-9598-2 20617376

[B21] Haupt RM Sings HL **The efficacy and safety of the quadrivalent human papillomavirus 6/11/16/18 vaccine gardasil** JAH 2011 14 5 467 475 10.1016/j.jadohealth.2011.07.003 22018560

[B22] Giuliano AR Palefsky JM Goldstone S Moreira ED Penny ME Aranda C Vardas E Moi H Jessen H Hillman R Chang YH Ferris D Rouleau D Bryan J Marshall JB Vuocolo S Barr E Radley D Haupt RM Guris D **Efficacy of quadrivalent HPV vaccine against HPV Infection and disease in males** N Engl J Med 2011 14 5 401 411 10.1056/NEJMoa0909537 21288094 PMC3495065

[B23] Mariani L Venuti A **HPV vaccine: an overview of immune response, clinical protection, and new approaches for the future** J Trans Med 2010 14 105 10.1186/1479-5876-8-105 PMC2988719 20979636

[B24] Korostil IA Peters GW Cornebise J Regan DG **Adaptive Markov chain Monte Carlo forward projection for statistical analysis in epidemic modelling of human papillomavirus** Stat Med 2013 14 11 1917 1953 10.1002/sim.5590 22961869

[B25] Burchell AN Coutlee F Tellier PP Hanley J Franco EL **Genital transmission of human papillomavirus in recently formed heterosexual couples** J Infect Dis 2011 14 11 1723 1729 10.1093/infdis/jir644 21984739 PMC3203235

[B26] Trottier H Franco EL **The epidemiology of genital human papillomavirus infection** Vaccine 2006 14 S1 15 16406226 10.1016/j.vaccine.2005.09.054

[B27] Trottier H Mahmud S Prado JCM Sobrinho JS Costa MC Rohan TE Villa LL Franco EL **TypeâSpecific duration of human papillomavirus infection: implications for human papillomavirus screening and vaccination** J Infect Dis 2008 14 1436 1447 18419547 10.1086/587698 PMC7889327

[B28] Rodriguez AC Schiffman M Herrero R Wacholder S Hildesheim A Castle PE Solomon D Burk R On behalf of the Proyecto Epidemiologico Guanacaste Group **Rapid clearance of human papillomavirus and implications for clinical focus on persistent infections** JNCI J Nat Cancer Inst 2008 14 7 513 517 10.1093/jnci/djn044 PMC3705579 18364507

[B29] Koshiol JE Schroeder JC Jamieson DJ Marshall SW Duerr A Heilig CM Shah KV Klein RS Cu-Uvin S Schuman P Celentano D Smith JS **Time to clearance of human papillomavirus infection by type and human immunodeficiency virus serostatus** Int J Cancer 2006 14 7 1623 1629 10.1002/ijc.22015 16646070

[B30] Giuliano AR Lu B Nielson CM Flores R Papenfuss MR Lee JH Abrahamsen M Harris RB **Age-specific prevalence, incidence, and duration of human papillomavirus infections in a cohort of 290 US men** J Infect Dis 2008 14 6 827 835 10.1086/591095 18657037

[B31] Giuliano AR Lee JH Fulp W Villa LL Lazcano E Papenfuss MR Abrahamsen M Salmeron J Anic GM Rollison DE Smith D **Incidence and clearance of genital human papillomavirus infection in men (HIM): a cohort study** Lancet 2011 14 9769 932 940 10.1016/S0140-6736(10)62342-2 21367446 PMC3231998

[B32] Garnett GP Anderson RM **Balancing sexual partnerships in an age and activity stratified model of HIV transmission in heterosexual populations** IMA J Math Appl Med Biol 1994 14 161 192 10.1093/imammb/11.3.161 7822888

[B33] Ferguson NM Garnett GP **More realistic models of sexually transmitted disease transmission dynamics: Sexual partnership networks, pair models, and moment closure** Sex Transm Dis 2000 14 10 600 10.1097/00007435-200011000-00008 11099075

[B34] **The national cervical screening program web site** [http://www.cancerscreening.gov.au/internet/screening/publishing.nsf/Content/cervical-about] last accessed: 25 July, 2013

[B35] **Australian bureau of statistics, 3101.0 - Australian demographic statistics** Sep 2012 [http://www.abs.gov.au/ausstats/abs@.nsf/mf/3101.0] last accessed: 26 August, 2013

[B36] Feinleib JA Michael RT **Reported changes in sexual behavior in response to AIDS in the United States** Prev Med 1998 14 3 400 411 10.1006/pmed.1998.0270 9612830

[B37] **Australian bureau of statistics, 3412.0 - Migration, Australia, 2010–11** [http://www.abs.gov.au/ausstats/abs@.nsf/detailsPage/3412.02010-11] last accessed: September 20, 2013

[B38] Olsson SE Villa LL Costa RLR Petta CA Andrade RP Malm C Iversen OE Høye J Steinwall M Riis-Johannessen G Andersson-Ellstrom A Elfgren K von Krogh G Lehtinen M Paavonen J Tamms GM Giacoletti K Lupinacci L Esser MT Vuocolo SC Saah AJ Barr E **Induction of immune memory following administration of a prophylactic quadrivalent human papillomavirus (HPV) types 6/11/16/18 L1 virus-like particle (VLP) vaccine** Vaccine 2007 14 26 4931 4939 10.1016/j.vaccine.2007.03.049 17499406

[B39] Plummer M Schiffman M Castle PE Maucort Boulch D Wheeler CM ALTS (Atypical Squamous Cells of Undetermined Significance/Low-Grade Squamous Intraepithelial Lesions Triage Study) Group **A 2-year prospective study of human papillomavirus persistence among women with a cytological diagnosis of atypical squamous cells of undetermined significance or low-grade squamous intraepithelial lesion** J Infect Dis 2007 14 11 1582 1589 10.1086/516784 17471427

