# Supplementary material for: The potential impact of HPV-16 reactivation on prevalence in older Australians
Source: BMC Infect Dis. 2014 Jun 6;14:312. doi: 10.1186/1471-2334-14-312 (PMC4061121; doi:10.1186/1471-2334-14-312)
Supplement: Additional file 1 — Technical Appendix. [file 1471-2334-14-312-S1.pdf]

The potential impact of HPV-16 reactivation on prevalence in older Australians
—
Technical Appendix

November 11, 2013

Contents

Assumptions regarding onset of menopause and reactivation 2
Demographic assumptions 6
Assumptions regarding vaccination 8
Model equations 9
Implementation of ageing 11
Implementation of sexual behaviour and calculation of the force of infection . . 12
Implementation of vaccination 13
Implementation of the onset of menopause 14
Specification of prior distributions for model parameters 15
Calibration stages 17
Likelihood function 18
Calibration plot 19

Assumptions regarding onset of menopause and reactivation

Assumption 1. *Onset of menopause occurs between 45 and 54.*

We arrived at this assumption based on two Australian studies which estimated that the median age of menopause in Australian women is 51 [6] and the mean age is 52.9 [13] (age range 45 to 64 years). Menopause in women closer to their 60s is quite rare. We, therefore, decided to allow for menopause in women aged 45-54.

We would like to stress that this is reasonable for Australia but not necessarily for any other country, because the age of natural menopause appears to be region dependent [4, 15].

Assumption 2. *All menopausal women who have been infected with HPV-16 at least once (i.e. who have been sufficiently exposed to HPV-16) are at risk of HVP-16 reactivation.*

According to [11], only whose menopausal women who have had at least 5 LTSPs appeared to be at risk of possible HPV reactivation, and there was no evidence that having more LTSPs increased the risk. This suggests that these women had a certain level of exposure to HPV achieved via having at least 5 LTSPs, and we speculate that this level is described as being infected at least once (in the absence of any other plausible explanations).

Assumption 3. *All women who have been infected with HPV-16 and become latently infected reactivate at menopause at age-dependent rates equal to the rates of onset of menopause.*

This is a simplification we see as appropriate in view of our limited knowledge of the reactivation process. The rates of onset of menopause are defined based on Australian data and discussed in “Implementation of the onset of menopause” on page 14.

Assumption 4. *Susceptible men at risk of reinfection with HPV-16 within the existing long-term partnerships (i.e. marriages or de facto) with 45-54 year old menopausal women at risk of reactivation are aged 45-54.*

No data on specific age differences in Australian couples in de facto partnerships are available (i.e. such data as what percentage of the 50 year old women are married to, for example, 53 year old men). However, it is acceptable to assume that an average 45-54 year old woman is likely to be in a de facto partnership with a 45-54 year old man. In most cases, according to ASHR [2], men would be expected to be older than their partners, but

given that ASHR effectively suggested the same sexual behaviour for individuals aged 45 to 59 (see also Table 2), we can claim that it doesn't matter to us whether for example, a 47 year old woman has a partner aged 48 or 55.

Assumption 5. *Reactivation of HPV-16 in Australian menopausal women should have started to occur on a relatively large scale in 2003.*

Year 2003 is when a fraction of 45 year old women who had a sufficient exposure to HPV-16 became significant enough to make the increased prevalence in these women noticeable. Please, see Figure 2 for details.

Assumption 6. *The masking effect of sexual revolution suggested by Gravitt et al. [11] is applicable to Australia.*

We assume this because Australia is a Western country in many respects similar to the US for which the effect has been originally discussed. The sexual revolution began in Australia right after it had begun in the US. The following reconstruction of sexual behaviour is based on the findings presented in [19].

pre-1961 While there is no comprehensive studies available on sexual behaviour in Australia before the sexual revolution, for the purpose of this study, it is sufficient to view it as varying around a level considerably lower than the one currently observed.

1961-1965 Year 1961 is a formal beginning of sexual revolution in Australia because that is when the contraceptive pill arrived in the county, a year after the US [1]. However, the pill was initially available only to married women and its use could not become widespread immediately. We, therefore, take 1966 as a the first year when noticeable changes in sexual behaviour could be observed.

1966-1975 This is a transition period signified by changes in sexual behaviour, perhaps, in women in the first place, and new attitudes towards sex in Australian society. Feminist movements were on the rise, sexual encounters between unmarried adults were losing their reprehensible public image, divorce rates were increasing and marriage rates were decreasing. All this was accompanied by the development of birth control and curability of all sexually transmitted diseases.

post-1976 While the notion of an "end" of sexual revolution is vague and lacking evidence, we assume that during this period there was no changes in sexual behaviour comparable in scale to those which happened during the Australian sexual revolution.

Figure 1 is a sketch intended to illustrate how the masking effect presumably works. The dashed line shows an age-specific HPV-16 prevalence profile in women before the sexual revolution. A much lower overall HPV prevalence and considerably more restrained sexual behaviour (as compared to the post-sexual revolution times) are attributed to

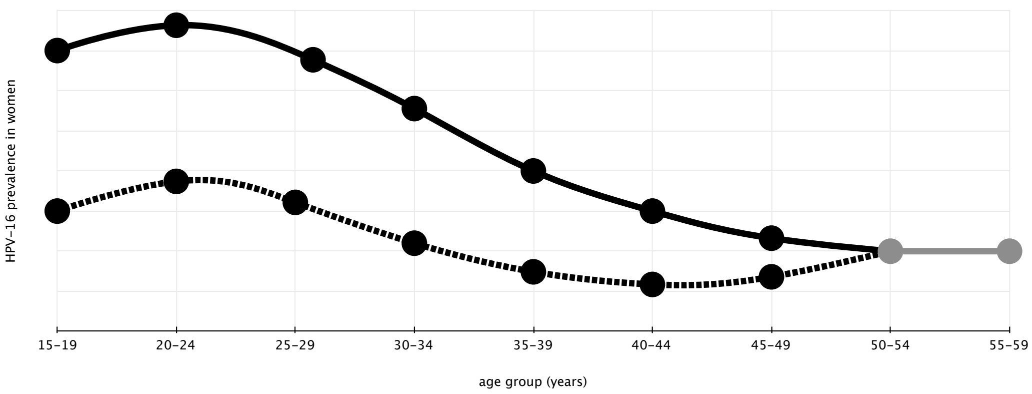

Figure 1: Masking effect of sexual revolution of 1961-1975 in Australia. Solid line marks age-specific prevalence observed around 2000; dashed line marks prevalence corresponding to pre-sexual revolution sexual behaviour - women with such behaviour were around their fifties at the time when prevalence was observed.

this period. The increased HPV prevalence in older women can be clearly identified. However, due to the sexual revolution and the following new sexual behaviour and high HPV prevalence (as compared with the pre-sexual revolution times), a cross-sectional study collecting HPV prevalence data in early 2000s would cover younger women with post-sexual revolution sexual experiences and older women whose sexual behaviour had been defined by the pre-sexual revolution norms. While there would be HPV reactivation in these older women, it would result in HPV-16 prevalence lower than that in the younger women from the post-sexual revolution cohort. Therefore, we would observe only the solid line which makes it impossible to see any signs of HPV reactivation.

Below we present a timeline which indicates the events important in the context of possible HPV reactivation in Australian women.

Assumption 7. *Probability of becoming latently infected (per detectable HPV-16 infection) does not depend on age.*

This is assumed for both men and women. Each time a woman of any age becomes infected with HPV-16, she may remain latently infected with probability p_w. Similarly, men can remain latently infected with probability p_m. This simplification has been made because there is currently no data or even plausible speculations suggesting age or any other factors the probabilities in question should depend upon.

It is pertinent to note that Assumption 7 allows us to justify estimation of both p_w and p_m via calibration of our model to the data for 15-39 year old women. If any HPV-16 prevalence data for Australian older women were available, estimations of these probabilities could be different from the ones we obtained.

Assumption 8. *Reactivation in menopausal women before 2003 can be seen as insignificant for the purposes of our study.*

We ignore reactivation in menopausal women before 2003. We ague that this is acceptable, given the purpose of our study, for the following reasons:

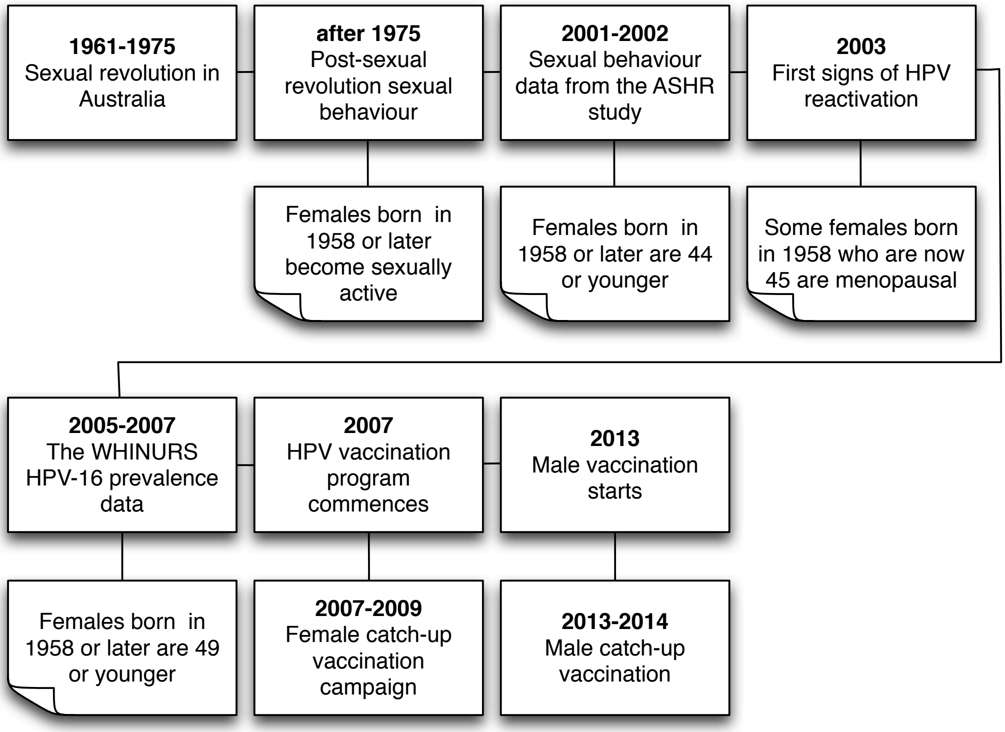

Figure 2: HPV-16 reactivation in the Australian setting. The ASHR data mentioned here are as in Table 2 and the WHINURS data can be found in Figure 11.

- The importance of exploration of the pre-2003 state of reactivation is questionable from the public health perspective.

Indeed, it is of interest to simulate the development of reactivation-related scenarios relevant now and in the near future.

- Allowing for reactivation of HPV-16 in menopausal women prior to 2003 would have a negligible effect on calibration of our model.

The available WHINURS data we use for calibration does not cover women over 39, so whatever HPV-16 prevalence the model produces for older women is not taken into account by the fitting procedure. Admittedly, an increased prevalence in menopausal women might influence prevalence in younger women used in the calibration procedure either via older men infected in partnerships with reactivating women and then infecting their new younger sexual partners, or via reactivating women acquiring new younger sexual partners who then pass the infection to their subsequent sexual partners. Both scenarios are not unlikely, but the ASHR sexual behaviour data suggest they are rather rare, so given that the data we use comes with wide confidence intervals, a slight increase in HPV prevalence in 15-39 year old women would have no dramatic consequences.

- Allowing for reactivation of HPV-16 in menopausal women prior to 2003 would require historical HPV-16 data which are not available in Australia.

We would be unable to properly estimate what women would be at risk of reactivation without historical sexual behaviour and HPV-16 prevalence data, which are not available for Australia. The ASHR data provide us with an estimate of the average number of sexual partners menopausal women (aged 45-54) have in 2001-2002, but we still need to know to what extent these women have been exposed to HPV when they were younger.

Demographic assumptions

We developed a dynamic compartmental model for HPV-16 transmission in the Australian heterosexual population. The population was stratified as shown in Figure 3.

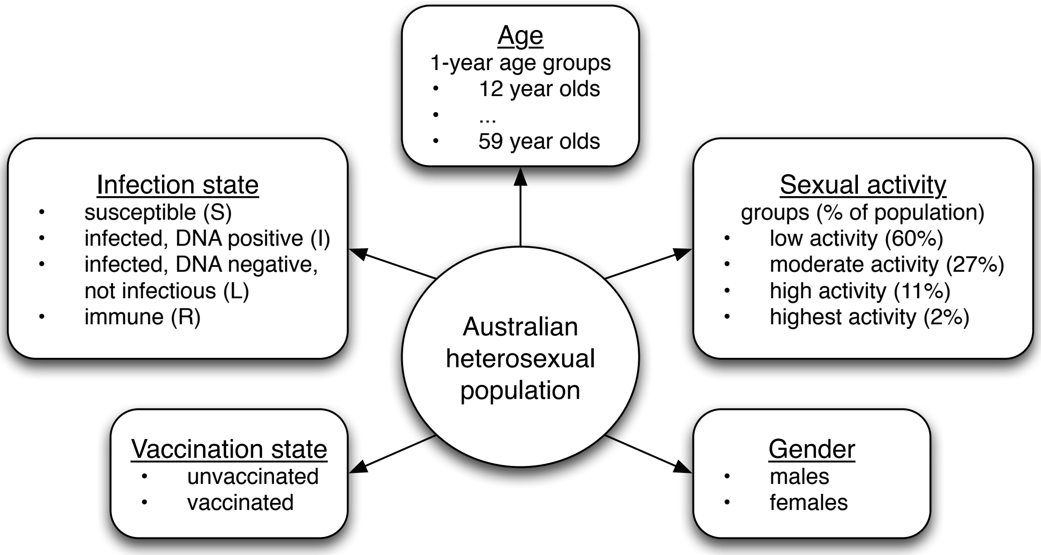

Figure 3: Stratification of the modelled population.

Assumption 9. *Australian sexually active population consists of 13-59 year old individuals.*

In our opinion, this is adequate for the purposes of our study for the following reasons:

1. In the context of HPV-16 reactivation, we are interested menopausal women aged 45-54, since we assume that other women who are older than 54 do not reactivate. Individuals aged 60 and older would certainly be of interest if we modelled HPV-16 related cancers, which we do not in this study.

2. The available sexual behaviour data (from the ASHR study) cover only individuals aged up to 59.

Assumption 10. *In each 1-year age group, namely, "12 year olds", "13 year olds", and so on to "59 year olds", the number of individuals is the same.*

This is not to be confused with the number of sexually active individuals in each age group! We think that this assumption is acceptable considering the available Australian data presented in Figure 4. Clearly, there is fewer 15-19 and 55-59 year old individuals in Australia, but considering the width of confidence intervals imposed on the HPV-16 prevalence data we calibrate our model to (see Figure 11), assuming that the 15-19 year olds constitute not about 8% but 8.5% of the entire population can be reasonably expected to have little impact on the calibration procedure.

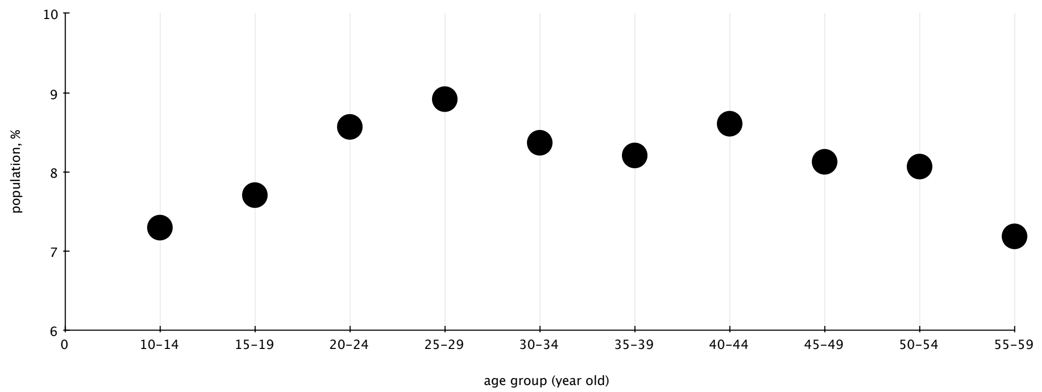

Figure 4: Australian population by age group, as reported by the Australian Bureau of Statistics [3].

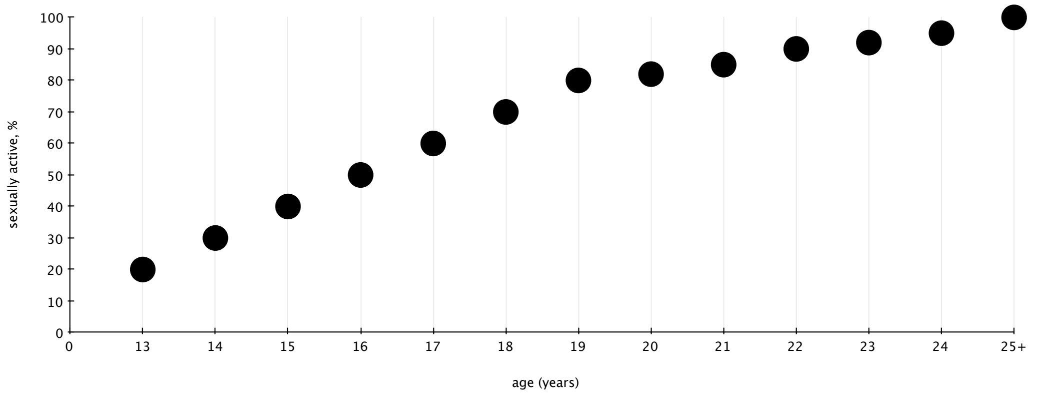

Figure 5: Percentage of sexually active in Australian population (no difference between males and females), see [7, 12, 26].

Assumption 11. *The number of males and females is the same in each 1-year age group.*

This simplification is reasonable because the available Australian data shows that the sex ratio indeed insignificantly varies around 1 between age groups and year to year.

Assumption 12. *For each 1-year age group, the proportion of sexually active men is the same as the proportion of sexually active women.*

This is based on the estimated age-specific proportions of sexually active individuals in Australian population shown in Figure 5 [7, 12].

Assumption 13. *Mortality due to HPV-16 or natural causes can be ignored, as well as population growth.*

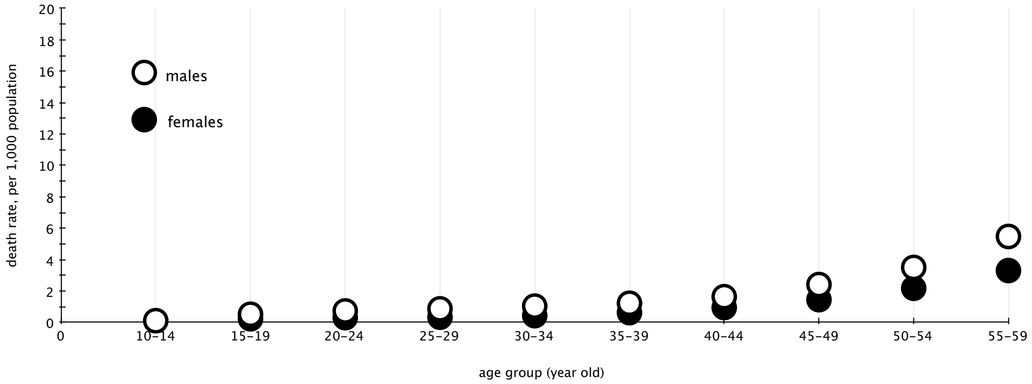

Figure 6: The age-specific death rates per 1,000 averaged over 10 years (2001-2011) used in our model.

According to the available data (Figure 6), death rates in the population of interest (12-59 year olds) do not appears substantial enough to have a notable effect on the magnitude of our estimations.

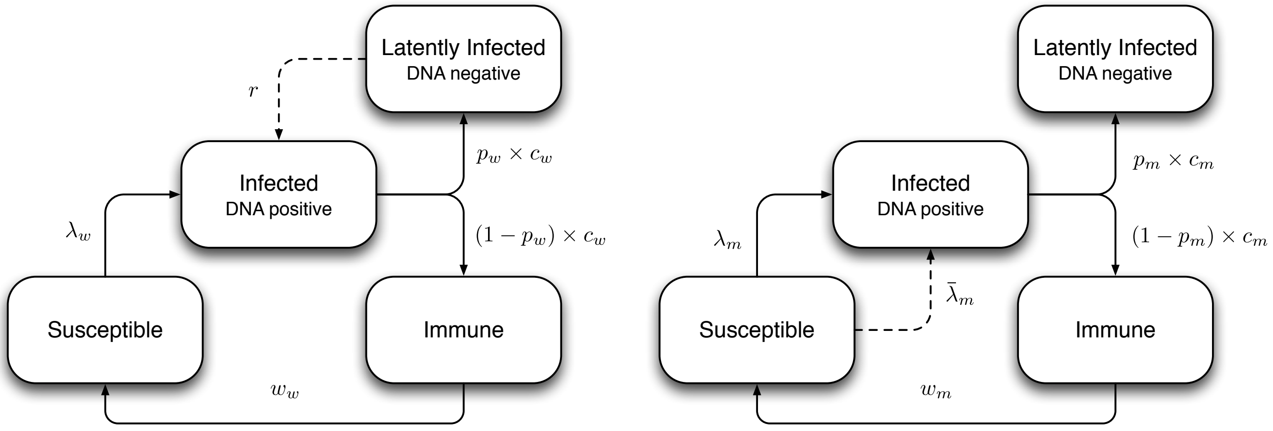

(a) Females. (b) Males.

Figure 7: HPV-16 transmission model.

Assumptions regarding vaccination

Assumption 14. *Duration of vaccine induced protection, expressed as reduced susceptibility to reinfection, is life-long.*

The validity of this assumption has been previously discussed (see, for example, [26]) and in brief, the argument to support it is that given the reported duration of vaccine protection exceeding eight years for young women, boys and girls [16, 24] and a possibility of a post-vaccination immune memory [20], it is reasonable to assume the life-long protection because even if it was not life-long as such, it could be maintained at an appropriate level by boosters.

model parameter	symbol	prior specification	refs.	posterior mean	SD
Natural history parameters					
probability of HPV-16 transmission					
from female to male	β_{wm}	$U(0.10, 1.00)$	[5]	0.92	0.03
from male to female	β_{mw}	$U(0.10, 1.00)$	[5]	0.93	0.03
rate of clearance of HPV-16					
for women under 30	c_{w1}	$U(0.50, 1.17)$	[27, 28, 23, 18]	0.59	0.05
for women over 30	c_{w2}	$U(0.50, 1.17)$	[27, 28, 23, 18]	0.60	0.05
for men under 30	c_{m1}	$U(0.55, 1.66)$	[10, 9]	0.62	0.03
for men over 30	c_{m2}	$U(0.55, 1.66)$	[10, 9]	0.63	0.03
probability of becoming latently infected					
for women	p_w	$U(0.01, 1.00)$	n/a	0.11	0.04
for men	p_m	$U(0.01, 1.00)$	n/a	0.09	0.05
rate of loss of immunity					
for women under 30	w_{w1}	$U(0.01, 2.00)$	n/a	1.65	0.16
for women over 30	w_{w2}	$U(0.01, 2.00)$	n/a	1.67	0.15
for men under 30	w_{m1}	$U(0.01, 2.00)$	n/a	1.78	0.14
for men over 30	w_{m2}	$U(0.01, 2.00)$	n/a	1.82	0.10
Sexual behaviour parameters					
degree of assortativity					
by age group	ε_a	$U(0.05, 0.95)$	n/a	0.27	0.22
by sexual activity group	ε_s	$U(0.05, 0.95)$	n/a	0.64	0.21

Table 1: Model parameters, their prior distributions (all uniform), posterior means and standard deviations (SD). Note that all parameters denote quantities averaged over the modelled population; the degrees of assortativity ε_a and ε_s are implemented as described in [8]; n/a indicates that there is no published literature to inform the choice of prior distribution and the values given are based on assumption. The choice of prior distributions is discussed in detail in Technical Appendix.

Model equations

Here we present the equations describing the movement between compartments in the sexually active population (note that 12 year olds are not sexually active!). All model equations are the ordinary differential equations. The notations we use are the following: infection states are S (susceptible), I (infected and HPV-16 DNA positive), L (infected and HPV-16 DNA negative, not infectious) and R (remover or immune); index s is for a sexual activity group ($s = 1, 2, 3, 4$), a is for an age group $12, 13, \ldots, 59$, v denotes vaccination status, not vaccinated $v-$ or vaccinated $v+$. Coefficients are as described in Table 1.

Susceptible

Women

$$\dot{S}_{w,s,[13-29],v-} = -\lambda_{w,s,[13-29],v-} S_{w,s,[13-29],v-} + w_{w1} R_{w,s,[13-29],v-}, \text{ unvaccinated under 30}$$

$$\dot{S}_{w,s,[30-59],v-} = -\lambda_{w,s,[30-59],v-} S_{w,s,[30-59],v-} + w_{w2} R_{w,s,[30-59],v-}, \text{ unvaccinated 30+}$$

$$\dot{S}_{w,s,[13-29],v+} = -(1-e_f)\lambda_{w,s,[13-29],v-} S_{w,s,[13-29],v+} + w_{w1} R_{w,s,[13-29],v+}, \text{ vaccinated under 30}$$

$$\dot{S}_{w,s,[30-59],v+} = -(1-e_f)\lambda_{w,s,[30-59],v-} S_{w,s,[30-59],v-} + w_{w2} R_{w,s,[30-59],v-}, \text{ vaccinated 30+}$$

Men

$$\dot{S}_{m,s,[13-29],v-} = -\lambda_{m,s,[13-29],v-}S_{m,s,[13-29],v-} + w_{m1}R_{m,s,[13-29],v-}, \text{ unvaccinated under 30}$$
$$\dot{S}_{m,s,[30-59],v-} = -\lambda_{m,s,[30-59],v-}S_{m,s,[30-59],v-} + w_{m2}R_{m,s,[30-59],v-}, \text{ unvaccinated 30+}$$
$$\dot{S}_{m,s,[13-29],v+} = -(1-e_m)\lambda_{m,s,[13-29],v-}S_{w,s,[13-29],v+} + w_{m1}R_{m,s,[13-29],v+}, \text{ vaccinated under 30}$$
$$\dot{S}_{m,s,[30-59],v+} = -(1-e_m)\lambda_{m,s,[30-59],v-}S_{w,s,[30-59],v-} + w_{m2}R_{m,s,[30-59],v-}, \text{ vaccinated 30+}$$

Infected (DNA positive)

Women

$$\dot{I}_{w,s,[13-29],v-} = -I_{w,[13-29],a,v-}c_{w1} + \lambda_{w,s,[13-29],v-}S_{w,s,[13-29],v-}, \text{ unvaccinated under 30}$$
$$\dot{I}_{w,s,[30-44],v-} = -I_{w,[30-44],a,v-}c_{w2} + \lambda_{w,s,[30-44],v-}S_{w,s,[30-44],v-}, \text{ unvaccinated 30-44}$$
$$\dot{I}_{w,s,[45-54],v-} = -I_{w,[45-54],a,v-}c_{w2} + \lambda_{w,s,[45-54],v-}S_{w,s,[45-54],v-} + I^*_{w,s,[45-54],v-}, \text{ unvaccinated 45-54}$$
$$\dot{I}_{w,s,[55-59],v-} = -I_{w,[55-59],a,v-}c_{w2} + \lambda_{w,s,[55-59],v-}S_{w,s,[55-59],v-}, \text{ unvaccinated 55-59}$$
$$\dot{I}_{w,s,[13-29],v+} = -I_{w,[13-29],a,v+}c_{w1} + (1-e_f)\lambda_{w,s,[13-29],v+}S_{w,s,[13-29],v+}, \text{ vaccinated under 30}$$
$$\dot{I}_{w,s,[30-59],v-} = -I_{w,[30-59],a,v-}c_{w2} + (1-e_f)\lambda_{w,s,[30-59],v-}S_{w,s,[30-59],v-}, \text{ vaccinated 30-59}$$

Men

$$\dot{I}_{m,s,[13-29],v-} = -I_{m,[13-29],a,v-}c_{m1} + \lambda_{m,s,[13-29],v-}S_{m,s,[13-29],v-}, \text{ unvaccinated under 30}$$
$$\dot{I}_{m,s,[30-44],v-} = -I_{m,[30-44],a,v-}c_{m2} + \lambda_{m,s,[30-44],v-}S_{m,s,[30-44],v-}, \text{ unvaccinated 30-44}$$
$$\dot{I}_{m,s,[45-54],v-} = -I_{m,[45-54],a,v-}c_{m2} + \lambda_{m,s,[45-54],v-}S_{m,s,[45-54],v-} + I^*_{m,s,[45-54],v-}, \text{ unvaccinated 45-54}$$
$$\dot{I}_{m,s,[55-59],v-} = -I_{m,[55-59],a,v-}c_{m2} + \lambda_{m,s,[55-59],v-}S_{m,s,[55-59],v-}, \text{ unvaccinated 55-59}$$
$$\dot{I}_{m,s,[13-29],v+} = -I_{m,[13-29],a,v+}c_{m1} + (1-e_m)\lambda_{m,s,[13-29],v+}S_{m,s,[13-29],v+}, \text{ vaccinated under 30}$$
$$\dot{I}_{m,s,[30-44],v+} = -I_{m,[30-44],a,v+}c_{m2} + (1-e_m)\lambda_{m,s,[30-44],v-}S_{m,s,[30-44],v+}, \text{ vaccinated 30-44}$$
$$\dot{I}_{m,s,[45-54],v+} = -I_{m,[45-54],a,v+}c_{m2} + (1-e_m)\lambda_{m,s,[45-54],v-}S_{m,s,[45-54],v+} + (1-e_m)I^*_{m,s,[45-54],v-},$$
$$\dot{I}_{m,s,[55-59],v+} = -I_{m,[55-59],a,v+}c_{m2} + (1-e_m)\lambda_{m,s,[55-59],v-}S_{m,s,[55-59],v+}, \text{ vaccinated 55-59}$$

Latently infected (DNA negative)

Women

$$\dot{L}_{w,s,[13-29],v-} = -p_w c_{w1} I_{w,[13-29],a,v-}, \text{ unvaccinated under 30}$$
$$\dot{L}_{w,s,[30-44],v-} = -p_w c_{w2} I_{w,[30-44],a,v-}, \text{ unvaccinated 30-44}$$
$$\dot{L}_{w,s,[45-54],v-} = -p_w c_{w2} I_{w,[45-54],a,v-} - I^*_{w,s,[45-54],v-}, \text{ unvaccinated 45-54}$$
$$\dot{L}_{w,s,[55-59],v-} = -p_w c_{w2} I_{w,[55-59],a,v-}, \text{ unvaccinated 55-59}$$
$$\dot{L}_{w,s,[13-29],v+} = -p_w c_{w1} I_{w,[13-29],a,v+}, \text{ vaccinated under 30}$$
$$\dot{L}_{w,s,[30-44],v+} = -p_w c_{w2} I_{w,[30-44],a,v+}, \text{ vaccinated 30-59}$$

Men

$$\begin{aligned}
\dot{L}_{m,s,[13-29],v-} &= -p_m c_{m1} I_{m,[13-29],a,v-}, \text{ unvaccinated under 30} \\
\dot{L}_{m,s,[30-59],v-} &= -p_m c_{m2} I_{m,[30-59],a,v-}, \text{ unvaccinated 30-59} \\
\dot{L}_{m,s,[13-29],v+} &= -p_m c_{m1} I_{m,[13-29],a,v+}, \text{ vaccinated under 30} \\
\dot{L}_{m,s,[30-44],v+} &= -p_m c_{m2} I_{m,[30-44],a,v+}, \text{ vaccinated 30-59}
\end{aligned}$$

Immune

Women

$$\begin{aligned}
\dot{R}_{w,s,[13-29],v-} &= -w_{w1} R_{w,s,[13-29],v-} + (1-p_w) c_{w1} I_{w,[13-29],a,v-}, \text{ unvaccinated under 30} \\
\dot{R}_{w,s,[30-59],v-} &= -w_{w2} R_{w,s,[30-59],v-} + (1-p_w) c_{w1} I_{w,[30-59],a,v-}, \text{ unvaccinated 30+} \\
\dot{R}_{w,s,[13-29],v+} &= (1-p_w) c_{w1} I_{w,[13-29],a,v+}, \text{ vaccinated under 30} \\
\dot{R}_{w,s,[30-59],v+} &= (1-p_w) c_{w1} I_{w,[30-59],a,v+}, \text{ vaccinated 30+}
\end{aligned}$$

Men

$$\begin{aligned}
\dot{R}_{m,s,[13-29],v-} &= -w_{m1} R_{m,s,[13-29],v-} + (1-p_m) c_{w1} I_{m,[13-29],a,v-}, \text{ unvaccinated under 30} \\
\dot{R}_{m,s,[30-59],v-} &= -w_{m2} R_{m,s,[30-59],v-} + (1-p_m) c_{w1} I_{m,[30-59],a,v-}, \text{ unvaccinated 30+} \\
\dot{R}_{m,s,[13-29],v+} &= (1-p_m) c_{m1} I_{m,[13-29],a,v+}, \text{ vaccinated under 30} \\
\dot{R}_{m,s,[30-59],v+} &= (1-p_m) c_{m1} I_{m,[30-59],a,v+}, \text{ vaccinated 30+}
\end{aligned}$$

Here $I^*_{w,s,[45-54],v-}$ and $I^*_{m,s,[45-54],v-}$ are terms due to inclusion of latency and reactivation into the model. The first one is for women and we define it as follows

$$\forall\, a \in \{45, 46, \ldots, 54\}\ I^*_{w,s,a,v-} = r_a L_{w,s,a,v-} \tag{1}$$

where r_a is a rate of reactivation specified as discussed in "Implementation of the onset of menopause" on page 14. Recall that reactivation in our model occurs simultaneously with the onset of menopause. For men,

$$\forall\, a \in \{45, 46, \ldots, 54\}\ I^*_{m,s,a,v-} = 0.7 \times S_{m,s,a,v-} \times \frac{r_a L^*_{w,s,a,v-}}{N_w} \times \beta_{wm}, \tag{2}$$

where 0.7 is the proportion of susceptible men who are in long-term relationships (i.e. married or de facto), and N_w is the total number of all women in the modelled population.

Implementation of ageing

Ageing in our model is discrete, which is why is is not a part of the model equations listed above. The way it works is based on a well known approach suggested in [25]:

- we solve the model equations on a time interval which corresponds to one year ($t \in [0, 1]$). This gives us the state of the model at the end of the current year;

- everyone in the model is *instantly* made older by 1 year; the number new 12 year old individuals who enter the model is equal to the number of individuals leaving the model;
- the obtained model state is used as the initial condition to solve the model equations on the time interval corresponding to the next year.

Implementation of sexual behaviour and calculation of the force of infection

HPV is sexually transmitted, so modelling of sexual behaviour within a population is a necessary part of any HPV model. In our study, we used the sexual behaviour data from a large cross sectional population survey the ASHR in Australia [2], a computer-assisted telephone survey of a random sample of about 20, 000 aged from 16 to 59.

The ASHR data was employed to fill a sexual mixing matrix, which is a matrix of conditional probabilities that an individual of a given age and sexual behaviour group would form a sexual partnership with an individual of the opposite gender of a particular age and sexual behaviour group.

**age group** year old	**sexual debut** (approx. year)		**sexual activity group (% of females)** 1 (60%)	2 (27%)	3 (11%)	4 (2%)
		RPC	1.00	4.76	24.83	105.65
16-19	after 1975	5.28	0.13	0.65	3.39	14.43
20-24	after 1975	6.06	0.15	0.74	3.89	16.56
25-29	after 1975	4.37	0.11	0.53	2.80	11.94
30-34	after 1975	2.57	0.06	0.31	1.65	7.02
35-39	after 1975	1.61	0.04	0.19	1.03	4.40
40-44	after 1975	1.43	0.03	0.17	0.91	3.90
45-49	1971-1975					
50-54	1966-1970	1.00	0.02	0.12	0.64	2.73
55-59	1961-1965					

Table 2: Age-specific annual sexual partner change rates for Australian females calculated based on the ASHR data and demographic data from Australian Bureau of Statistics for June, 2002. RPC is relative sexual partner change rate by age or sexual activity group derived in [21]. The overall population sexual partner change rate is 0.437.

Following the notations used in [29], let g denote a gender, and g' an opposite gender; a and a' are some age groups, and s and s' are some sexual activity groups; $P_{g'as}$ is the number of partnerships generated by people of gender g' in age group a and sexual activity group s. Then, according to the formulation introduced in [8], the probability that someone of gender g in age group a and sexual activity group s will form a partnership with someone of gender g' in age group a' and sexual activity group s' is defined as

$$\rho_{gasa's'} \equiv \left(\varepsilon_a \delta_{aa'} + (1-\varepsilon_a) \frac{\sum_{\beta=1}^{n_s} P_{g'a'\beta}}{\sum_{\alpha=1}^{n_A} \sum_{\beta=1}^{n_s} P_{g'\alpha\beta}} \right) \times \left(\varepsilon_s \delta_{ss'} + (1-\varepsilon_s) \frac{\sum_{\beta=1}^{n_s} P_{g'a's'}}{\sum_{\beta=1}^{n_s} P_{g'a'\beta}} \right) \quad (3)$$

where ε_a and ε_s are the degrees of assortativity by age and sexual activity group, and

$$\delta_{aa'} = \begin{cases} 1, & a = a' \\ 0, & a \neq a' \end{cases}$$

Note that if $\varepsilon_a = 0$, the first factor in the product is simply the proportion of all partnership generated by people of gender g' attributed to the age group j, and sexual mixing is called proportional by age. If $\varepsilon_a = 1$, then the first factor is non-zero only if $a = a'$, which means that a partnership can be established strictly between people from the same age group. Then sexual mixing is called fully assortative by age. Similar considerations are valid for the second factor, representing mixing by sexual activity.

As recently discussed in [29], formulation (3) is widely used but not entirely correct, and it is more appropriate to define ρ_{kihjm} as below.

$$\rho_{gasa's'} \equiv \varepsilon_a \varepsilon_s \delta_{ij} \delta_{ss'} + \varepsilon_a (1-\varepsilon_s) \frac{P_{g'a's'}}{\sum_{\beta=1}^{n_s} P_{g'j\beta}} \delta_{aa'} + \\ (1-\varepsilon_a)\varepsilon_s \frac{P_{g'a's'}}{\sum_{\alpha=1}^{n_A} P_{g'\alpha s'}} \delta_{ss'} + (1-\varepsilon_a)(1-\varepsilon_s) \frac{P_{g'a's'}}{\sum_{\alpha=1}^{n_A}\sum_{\beta=1}^{n_s} P_{g'\alpha\beta}}. \quad (4)$$

The degrees of assortativity are very hard to determine based on the results of sexual behaviour surveys, no matter that some reasonable assumptions regarding them may be made. For example, one might assume that ε_a is higher for those attending school than for other people, because because their social contacts are largely limited to their own age group; or, ε_s might be low for the most sexually active group because this group may mainly include sex workers servicing less active clients. However, such assumptions are rather speculative and rarely backed by sufficient data. Therefore, it is beneficial to consider ε_a and ε_s as model parameters with their own prior distributions assigned.

To fill the matrix we used the data presented in Table 2, which originally was extracted from the findings of Australian Study of Health and Relationships (ASHR) [2] in [21]. These data are relative sexual partner acquisition rates for each age group a (we denote them r_a) and each sexual activity (risk) group s (r_s). The overall sexual partner change rate $\bar{c}$ averaged over the entire population was fixed at 0.437. We should emphasise the assumption that there is no difference in sexual activity between females and males from the same group.

In order to calculate the force of infection, we follow the scheme outlined in [8].

Remark on initiation of sexual activity

In our model, sexual activity commences instantaneously on yearly basis. Suppose at the end of a year N_{17} of the 17 year olds have to become the 18 year olds, and the proportion of sexually active in 17 year olds, $p_{17} \neq 1$, must become the proportion of sexually active in the 18 year olds, p_{18}. Since we store the sexually inactive in the susceptible compartment, this means that after the 17 year olds update their age label to 18, we have to move the fraction $(p_{18} - p_{17})/(1 - p_{17})$ of susceptible and sexually inactive to susceptible sexually active.

Implementation of vaccination

Suppose the plan is to vaccinate a fraction C_1 of N females from age group a during the first year of the catch-up campaign, and then further increase their coverage to C_2

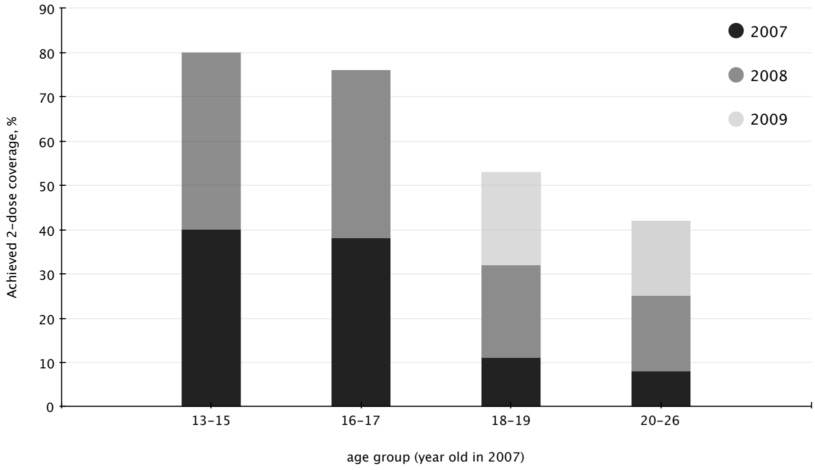

Figure 8: Australian HPV vaccination catch-up campaign of 2007-2009. The eligible females were those aged 13-26 in 2007. The bars show a percentage of these females vaccinated each year.

during the second year. After the first year we will have C_1N vaccinated females and $(1 - C_1)N$ unvaccinated ones. Next year, we vaccinate a fraction $(C_2 - C_1)/(1 - C_1)$ of these unvaccinated females to achieve the planned number of vaccinated females

$$\frac{C_2 - C_1}{1 - C_1}(1 - C_1)N + C_1N = C_2N.$$

Implementation of the onset of menopause

The percentages of women who reach menopause at each age from 45 to 54 are shown in Figure 9. These are based on the results presented in [6]. In the model, we assume that

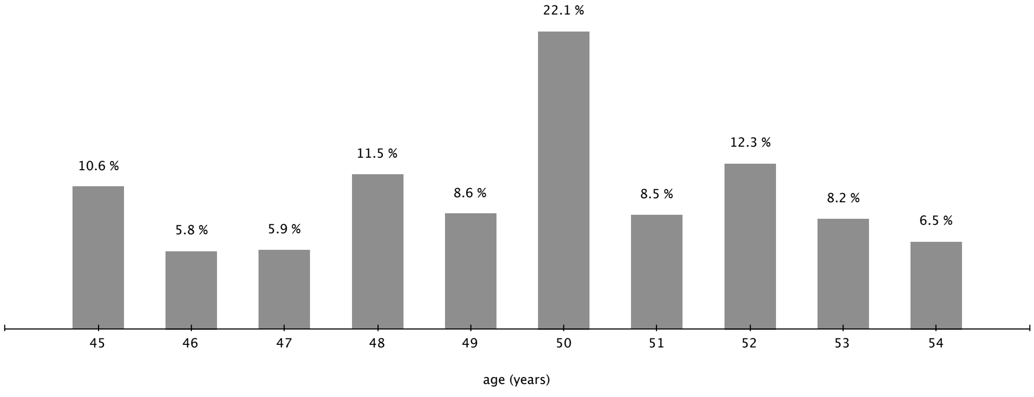

Figure 9: Age-specific onset of menopause for a cohort of Australian women as reported in [6]. For each age, the shown percents are percents of women who become menopausal at that age.

menopause occurs at a constant rate specific for each age. For example, suppose we have N women who turn 45. We know that $p_{45} = 0.106$ of them have to become menopausal before they are 46. Let r_{45} be a yearly rate at which this happens, and M be the number of women who have already become menopausal. Then for a year starting at $t = 0$ and

ending at $t = 1$ we can write down the following equation:

$$\begin{aligned} \dot{M}(t) &= r_{45}(N - M(t)), \\ M(t=0) &= 0, \\ M(t=1) &= p_{45} \times N. \end{aligned} \tag{5}$$

It is easy to see that this equation has a solution $M(t) = N + \exp(-r_{45}t)c_1$, where c_1 is a constant. We can insert this solution into the boundary conditions and obtain that $N + c_1 = 0$, $N + \exp(-r)c_1 = p_{45} \times N$. Hence, $r_{45} = -\ln(1 - p_{45})$. Next year, the women who have not become menopausal when they were 45 (there are $N - p_{45}N = (1 - p_{45})N$ of such women), become menopausal at a rate r_{46}. This rate is such that at the end of the year we will have $p_{46}N$ 46 year old women who have become menopausal during the year. So,

$$\begin{aligned} \dot{M}(t) &= r_{46}((1 - p_{45})N - M(t)), \\ M(t=0) &= 0, \\ M(t=1) &= p_{46} \times N. \end{aligned} \tag{6}$$

Then

$$p_{46} = -\ln\left(1 - \frac{p_{46}}{1 - p_{45}}\right).$$

In general, we have that

$$\forall a \in \{45, \ldots, 54\}\ p_a = -\ln\left(1 - \frac{p_a}{1 - \sum_{j=1}^{j=a-1} p_j}\right). \tag{7}$$

Specification of prior distributions for model parameters

Probability of HPV-16 transmission per partnership

This probability has been estimated at 0.20 (95% CI, 0.16-0.24) in [5] based on the findings of the HITCH Study (HPV Infection and Transmission among Couples through Heterosexual activity). There were 179 discordant couples enrolled (18-24 y.o. women and their partners). No notable differences between the probabilities of women-to-men (β_{wm}) and men-to-women (β_{mw}) transmissions have been detected. Some other studies, however, reported that women-to-men transmission probability was much higher. In particular, men-to-women transmission was was 4.5 per 100 person-months (95% CI, 1.5-9.3), while women-to-men transmission was 27.8 per 100 person-months (95% CI, 19.0-38.3) [14]. Only 25 couples were enrolled. Another study conducted in California also enrolled 25 couples (the average age was 25 years for males and 23 years for females), and the men-to-women transmission was 4.9 per 100 person-months (95% CI, .6-17.7), while the women-to-men transmission was 16.5 (95% CI, 6.6-33.9), both estimated over a 6-week period [30].

Taking into account a considerable uncertainty surrounding this probability, especially, in the context of compartmental models where all partnerships are treated as instantaneous, we decided to use the reported estimation to define only the lower boundary of the prior, that is, choose a uniform distribution U(0.1,1.0) for both β_{wm} and β_{mw}.

Mean rate of clearance of HPV-16 infection

We approximate the mean rate of clearance of HPV-16 infection as the inverse of the mean duration of HPV-16 infection.

The Ludwig-McGill cohort study enrolled 2528 women aged 18-60 years attending a maternal and child health program in São Paulo, Brazil, These women were followed for up to 10 years. Mean duration of HPV-16 infection was estimated at 11.9 months (95% CI: 10.3-13.5), which is 0.99 years (95% CI: 0.85-1.12) [28]. Importantly, age did not influence duration of infection.

Female university students (621 in total, ages 17-42, mean age 23) in Montreal were followed for 2 years at 6-month intervals [22]. The reported mean duration of infection was 18.3 (95% CI: 12.9-23.7), or in years, 1.52 (95% CI: 1.07-1.97).

The HERS cohort of 871 HIV-seropositive and 439 HIV-seronegative women enrolled at 4 sites in the US was followed for 4.4 years (median). It was established that the median duration of infection was 1 year, and there was no difference in duration between women aged 35 or less and women over age 35 [18] .

The Guanacaste study covering 10,049 women over 18, [23] reported that females under 30 tended to clear HPV infection quicker than females older that 30. For example, by 1 year the fraction of females who cleared infection were around 56% and 48%, respectively.

In view of the above, we introduced two parameters for the mean duration of infection, for females under 30 and over 30, with the same fairly wide prior uniform distribution $U(0.85, 2.0)$. This would allow for the possibility that the durations are age dependent.

In a prospective cohort study of 290 men aged 18-44 years, participants were examined at baseline and every 6 months, with a mean duration of follow-up of 15.5 months [10]. Median time to clearance of HPV-16 was 6.0 months (95% CI: 5.2-6.8), which is in years 0.5 (95% CI: 0.43-0.56).

The HPV in Men (HIM) study enrolled 4,074 men aged 18-70 years from Brazil, Mexico, and the USA [9]. They were assessed every 6 months for a median follow-up of 275 months. Median time to clearance of HPV-16 was 12.19 months (95% CI: 7.16-18.17), or in years, 1.01 (95% CI: 0.59-1.51). Median time to clearance of infection of any HPV type was significantly longer in men aged 18-30 years than in the other age groups. However, median time to clearance of HPV 16 was not age dependent.

Although we did not have any information about the mean durations of infection for males, we assumed that the mean would be greater than median as it was the case for females. Again, as we did for females, we introduced two duration of infection parameters for males under and over 30, with the same uniform prior distribution $U(0.6, 1.8)$. Any calibration induced differences between these parameters would be evident from their posterior distributions.

Probability to become latently infected

We had no information about this parameter, so its prior distribution was set to $U(0.01, 1.00)$. Note that in case it is approaching 0, both latency and reactivation in the model are negligible. If it is 1, all women or men who "clear" infection are actually latently infected, and clearance as such is non-existent.

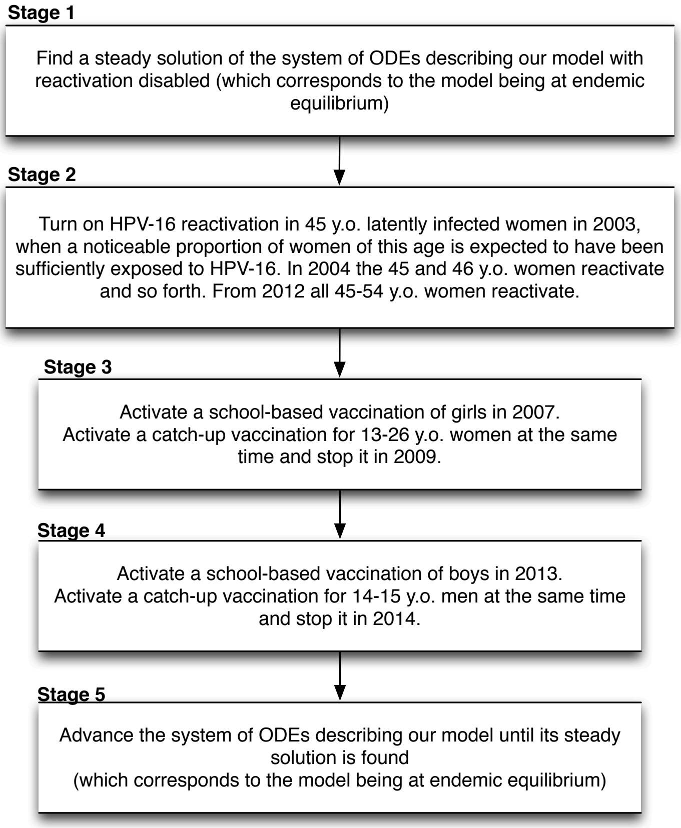

Figure 10: The stages of calibration procedure performed for each sampled parameter vector.

Sexual mixing parameters

Prior distributions for these parameters were selected based on the discussion in [21]. We are not aware of any conclusive data which would help to define priors for these parameters though, which is why we simply choose $U(0.1, 0.9)$ for both.

Calibration stages

The stages involved in a calibration procedure we use (detailed in [17]) are shown in Figure 10.

(1) We obtain an endemic equilibrium solution of the system describing our model with reactivation turned off, according to Assumption 8 (i.e. $r = 0$; note that $p \neq 0$ as women of all ages may enter the latently infected compartment). This gives us the numbers of individuals that should be in every model compartment given Australian sexual behaviour and the chosen parameter values. These numbers do not change in time. There is no ongoing vaccination in the model at this stage.

(2) A school-based vaccination of girls commences in 2007, along with a catch-up campaign for 13-26 year old women which lasts until 2009.

(3) HPV-16 reactivation is enabled in females aged 45 in 2008, because it is assumed that these women will already be from the post-sexual revolution cohort.

(4) A school-based vaccination of boys commences in 2013.along with a catch-up campaign for 14-15 year old boys which lasts until 2015.

(5) The model evolves until it reaches an endemic equilibrium when HPV-16 prevalence does not change in time anymore.

Likelihood function

While the calibration procedure is comprehensively described in [17], we describe here the choice of the likelihood function which is one of the key parts of the procedure.

The likelihood model we used is as follows. We assume that HPV-16 DNA prevalence P_a for each age group $a \in \overline{1,6}$ reported by the WHINURS study is drawn from a Beta distribution with shape parameters α_a and β_a. We will denote this distributions as Beta(α_a, β_a). Let the simulated HPV-16 DNA prevalence for age group a be P_a^*. We would like to choose parameters α_a and β_a so that the corresponding Beta distribution has mean at P_a^*. Since we know that the expression for mean of a Beta distribution is $\alpha_a/(\alpha_a + \beta_a)$, we can equate it to P_a^* and obtain that

$$\beta_a = \alpha_a \left(\frac{1}{P_a^*} - 1 \right).$$

Hence, we can define a log-likelihood function for the given data as follows,

$$\log \left\{ \prod_{a=1}^{6} \text{betapdf} \left(P_a, \alpha_a, \alpha_a \left(\frac{1}{P_a^*} - 1 \right) \right) \right\} = \sum_{a=1}^{6} \log \left\{ \text{betapdf} \left(P_a, \alpha_a, \alpha_a \left(\frac{1}{P_a^*} - 1 \right) \right) \right\},$$

where

$$\text{betapdf} \left(P_a, \alpha_a, \alpha_a \left(\frac{1}{P_a^*} - 1 \right) \right)$$

is a probability density function of Beta distribution evaluated at P_a.

Calibration plot

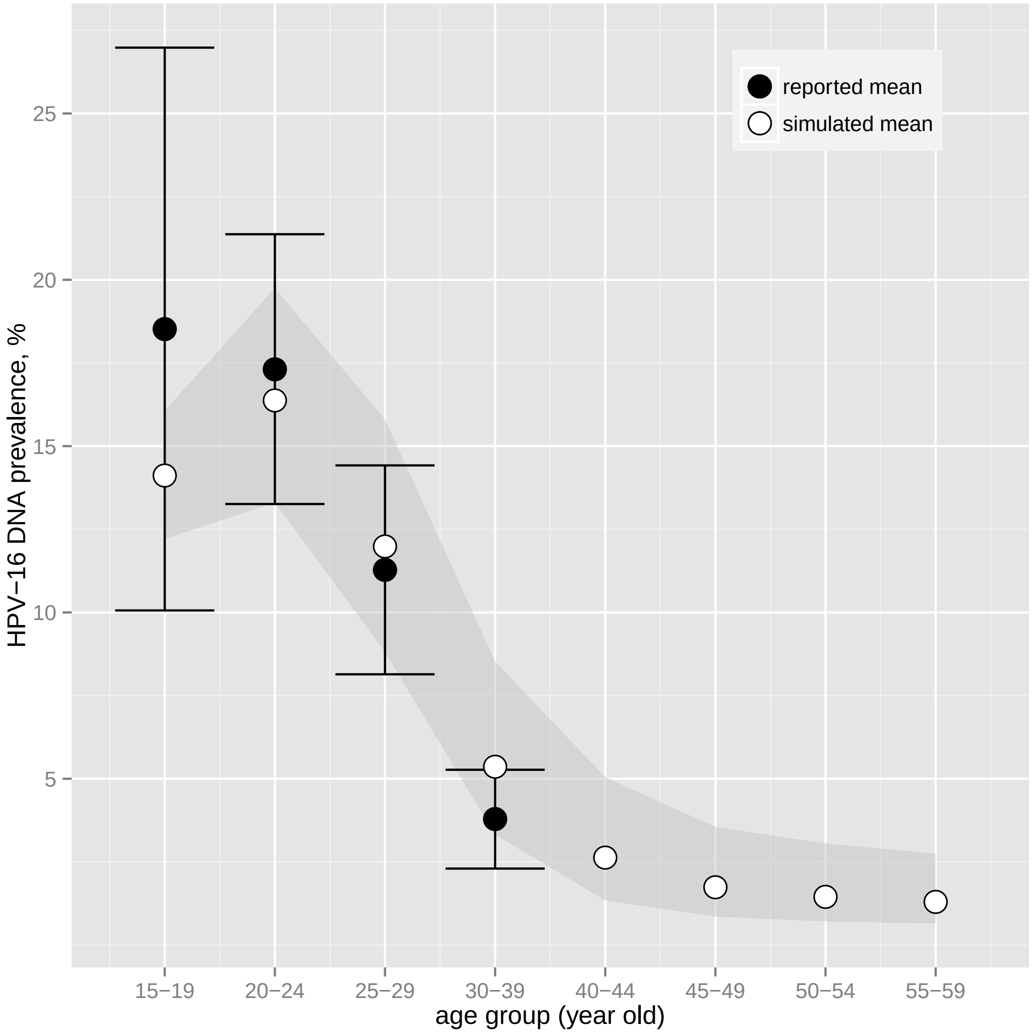

Figure 11: Claibration to the WHINURS data. The 95% confidence interval (grey area) is defined as an area between the 97.5-th and 2.5-th percentiles.

References

[1] The sexual revolution turns 50, The Sydney Morning Herald, May 11, 2010. http://www.smh.com.au/lifestyle/life/the-sexual-revolution-turns-50-20100511-uqj2.html.

[2] The Australian Study of Health and Relationships web site, http://www.ashr.edu.au, last accessed: 25 July, 2013.

[3] Australian Bureau of Statistics, 3101.0 - Australian Demographic Statistics, Sep 2012; http://www.abs.gov.au/ausstats/abs@.nsf/mf/3101.0, last accessed: 26 August, 2013.

[4] D J Brambilla and S M McKinlay. A prospective study of factors affecting age at menopause. *Journal of clinical epidemiology*, 42(11):1031–1039, 1989.

[5] Ann N Burchell, Francois Coutlee, Pierre-Paul Tellier, James Hanley, and Eduardo L Franco. Genital transmission of human papillomavirus in recently formed heterosexual couples. *The Journal of infectious diseases*, 204(11):1723–1729, December 2011.

[6] K A Do, S A Treloar, N Pandeya, D Purdie, A C Green, A C Heath, and N G Martin. Predictive factors of age at menopause in a large Australian twin study. *Human biology*, 70(6):1073–1091, December 1998.

[7] M P Dunne, M Donald, J Lucke, R Nilsson, R Ballard, and B Raphael. Age-related increase in sexual behaviours and decrease in regular condom use among adolescents in Australia. *Int J STD AIDS*, 5(1):41–47, 1994.

[8] Geoffrey P Garnett and Roy M Anderson. Balancing sexual partnerships in an age and activity stratified model of HIV transmission in heterosexual populations. *IMA journal of mathematics applied in medicine and biology*, 11:161–192, 1994.

[9] Anna R Giuliano, Ji Hyun Lee, William Fulp, Luisa L Villa, Eduardo Lazcano, Mary R Papenfuss, Martha Abrahamsen, Jorge Salmeron, Gabriella M Anic, Dana E Rollison, and Danelle Smith. Incidence and clearance of genital human papillomavirus infection in men (HIM): a cohort study. *Lancet*, 377(9769):932–940, March 2011.

[10] Anna R Giuliano, Beibei Lu, Carrie M Nielson, Roberto Flores, Mary R Papenfuss, Ji Hyun Lee, Martha Abrahamsen, and Robin B Harris. AgeSpecific Prevalence, Incidence, and Duration of Human Papillomavirus Infections in a Cohort of 290 US Men. *Journal of Infectious Diseases*, 198(6):827–835, September 2008.

[11] P E Gravitt, A F Rositch, M I Silver, M A Marks, K Chang, A E Burke, and R P Viscidi. A Cohort Effect of the Sexual Revolution May Be Masking an Increase in Human Papillomavirus Detection at Menopause in the United States. *Journal of Infectious Diseases*, 207(2):272–280, December 2012.

[12] A C Grunseit and J Richters. Age at first intercourse in an Australian national sample of technical college students. *Australian and New Zealand journal of public health*, 24(1):11–16, February 2000.

[13] J.R. Guthrie, L. Dennerstein, J.R. Taffe, P. Lehert, and H.G. Burger. Hot flushes during the menopause transition: a longitudinal study in Australian-born women. *Menopause*, 12(4):460–467, 2005.

[14] Brenda Y Hernandez, Lynne R Wilkens, Xuemei Zhu, Pamela Thompson, Katharine McDuffie, Yurii B Shvetsov, Lori E Kamemoto, Jeffrey Killeen, Lily Ning, and Marc T Goodman. Transmission of human papillomavirus in heterosexual couples. *Emerging infectious diseases*, 14(6):888–894, June 2008.

[15] I Kato, P Toniolo, A Akhmedkhanov, K L Koenig, R Shore, and A Zeleniuch-Jacquotte. Prospective study of factors influencing the onset of natural menopause. *Journal of clinical epidemiology*, 51(12):1271–1276, December 1998.

[16] Susanne Krüger Kjaer. Long-term effectiveness of Gardasil in the Nordic countries. Poster at the 28th International Papillomavirus Conference, Puerto Rico, 2012.

[17] Igor A Korostil, Gareth W Peters, Julien Cornebise, and David G Regan. Adaptive Markov chain Monte Carlo forward projection for statistical analysis in epidemic modelling of human papillomavirus. *Statistics in Medicine*, September 2012.

[18] Jill E Koshiol, Jane C Schroeder, Denise J Jamieson, Stephen W Marshall, Ann Duerr, Charles M Heilig, Keerti V Shah, Robert S Klein, Susan Cu-Uvin, Paula Schuman, David Celentano, and Jennifer S Smith. Time to clearance of human papillomavirus infection by type and human immunodeficiency virus serostatus. *International Journal of Cancer*, 119(7):1623–1629, 2006.

[19] Milton James Lewis. *Thorns on the Rose: the History of STDs in Australia in International Perspective*. Australian Govt. Pub. Service, 1998.

[20] Sven-Eric Olsson, Luisa L Villa, Ronaldo L R Costa, Carlos A Petta, Rosires P Andrade, Christian Malm, Ole-Erik Iversen, John Høye, Margareta Steinwall, Grete Riis-Johannessen, Agneta Andersson-Ellstrom, Kristina Elfgren, Geo von Krogh, Matti Lehtinen, Jorma Paavonen, Gretchen M Tamms, Katherine Giacoletti, Lisa Lupinacci, Mark T Esser, Scott C Vuocolo, Alfred J Saah, and Eliav Barr. Induction of immune memory following administration of a prophylactic quadrivalent human papillomavirus (HPV) types 6/11/16/18 L1 virus-like particle (VLP) vaccine. *Vaccine*, 25(26):4931–4939, June 2007.

[21] David G Regan, David J Philp, Jane S Hocking, and Matthew G Law. Modelling the population-level impact of vaccination on the transmission of human papillomavirus type 16 in Australia. *Sex. Health*, 4:147–163, 2007.

[22] Harriet Richardson, Gail Kelsall, Pierre Tellier, Hélène Voyer, Michal Abrahamowicz, Alex Ferenczy, Francois Coutlee, and Eduardo L Franco. The natural history of type-specific human papillomavirus infections in female university students. *Cancer Epidemiol Biomarkers Prev*, 12(6):485–490, June 2003.

[23] A C Rodriguez, M Schiffman, R Herrero, S Wacholder, A Hildesheim, P E Castle, D Solomon, R Burk, and On behalf of the Proyecto Epidemiologico Guanacaste Group. Rapid Clearance of Human Papillomavirus and Implications for Clinical Focus on Persistent Infections. *JNCI Journal of the National Cancer Institute*, 100(7):513–517, April 2008.

[24] Alfred Saah. Long-term extension study of Gardasil in adolescents; results through month 96. Poster at the 28th International Papillomavirus Conference, Puerto Rico, 2012.

[25] Dieter Schenzle. An Age-Structured Model of Pre- and Post-Vaccination Measles Transmission. *Mathematical Medicine and Biology*, 1(2):169–191, 1984.

[26] Megan A Smith, Karen Canfell, Julia M L Brotherton, Jie-Bin Lew, and Ruanne V Barnabas. The predicted impact of vaccination on human papillomavirus infections in Australia. *International Journal of Cancer*, 123(8):1854–1863, October 2008.

[27] Helen Trottier and Eduardo L Franco. The epidemiology of genital human papillomavirus infection. *Vaccine*, 24 Suppl 1:S1–15, March 2006.

[28] Helen Trottier, Salaheddin Mahmud, José Carlos M Prado, Joao S Sobrinho, Maria C Costa, Thomas E Rohan, Luisa L Villa, and Eduardo L Franco. TypeSpecific Duration of Human Papillomavirus Infection: Implications for Human Papillomavirus Screening and Vaccination. *Journal of Infectious Diseases*, 197(10):1436–1447, May 2008.

[29] Robert Walker, Carolyn Nickson, Jie-Bin Lew, Megan Smith, and Karen Canfell. A revision of sexual mixing matrices in models of sexually transmitted infection. *Statistics in Medicine*, pages n/a–n/a, July 2012.

[30] Lea E Widdice, David J Breland, Janet Jonte, Sepideh Farhat, Yifei Ma, Anthony C Leonard, and Anna-Barbara Moscicki. Human Papillomavirus Concordance in Heterosexual Couples. *Journal of Adolescent Health*, 47(2):151–159, August 2010.
